# Treatment options for patients with human epidermal growth factor 2-positive breast cancer brain metastases: A systematic review and meta-analysis

**DOI:** 10.3389/fonc.2023.1003565

**Published:** 2023-02-20

**Authors:** Xingfa Huo, Guoshuang Shen, Tianzhuo Wang, Jinming Li, Qiqi Xie, Zhen Liu, Miaozhou Wang, Fuxing Zhao, Dengfeng Ren, Jiuda Zhao

**Affiliations:** ^1^ Breast Disease Diagnosis and Treatment Center of Affiliated Hospital of Qinghai University & Affiliated Cancer Hospital of Qinghai University, Xining, China; ^2^ Precision Medicine Center of Oncology, The Affiliated Hospital of Qingdao University, Qingdao, Shandong, China

**Keywords:** treatment, HER2, breast cancer, brain metastases, meta-analysis

## Abstract

**Introduction:**

Many systemic treatment options are available for patients with human epidermal growth factor 2 (HER2)-positive breast cancer brain metastases. However, it is unclear which pharmacological treatment option is the most beneficial.

**Methods:**

We searched databases, such as PubMed, Embase, and Cochrane Library, and conference abstracts according to keywords. We extracted progression-free survival (PFS), overall survival (OS) data, and overall response rate (ORR) from randomized controlled trials and single-arm studies of HER2-positive breast cancer brain metastasis treatment for meta-analysis and analyzed different drug-related adverse events (AEs).

**Results:**

Three randomized controlled trials and seven single-arm clinical studies with 731 patients with HER2-positive brain metastases from breast cancer involving at least seven drugs were included. In randomized controlled trials, our results showed that trastuzumab deruxtecan significantly improved PFS and OS in patients and was superior to other drug regimens. In the single-arm study, the ORR was more pronounced for the trastuzumab deruxtecan and pyrotinib plus capecitabine regimens (ORR, 73.33%; 95% confidence intervals [CI], 44.90%−92.21%; ORR, 74.58%; 95% CI, 61.56%−85.02%, respectively). We found that the main AEs of antibody-drug conjugate (ADC) were nausea and fatigue, while the main AE of small-molecule tyrosine kinase inhibitor (TKI) drugs and large monoclonal antibodies was diarrhea.

**Conclusions:**

Trastuzumab deruxtecan was shown to be the most significant in improving survival in patients with HER2-positive breast cancer brain metastases in network meta-analysis, and in single-arm study, patients with HER2-positive breast cancer brain metastases treated with trastuzumab deruxtecan and pyrotinib plus capecitabine regimen had the highest ORR. The main AEs associated with ADC, large monoclonal antibodies, and TKI drugs were nausea, fatigue, and diarrhea, respectively.

## Introduction

The latest epidemiological data show that breast cancer brain metastases have the second highest incidence after non-small cell lung cancer brain metastases among primary tumors (1–3). The incidence of brain metastases in human epidermal growth factor 2 (HER2)-positive and triple-negative breast cancers ranges from 30% to 55% and 25% to 46%, respectively, and HER2-positive breast cancers have the highest incidence of brain metastases. HER2-positive breast cancer brain metastases occur late and usually occur in advanced stages of the disease (4–6). Brain metastases from breast cancer are significantly associated with a shorter survival, and the average overall survival (OS) of patients with brain metastases is approximately 2−6 months (7). To date, the treatment of breast cancer brain metastasis is mainly local treatment, supplemented by systemic treatment, including surgery, whole-brain radiation therapy, stereotactic radiation therapy (SRS), other local treatments, and drug treatment for some special types of brain metastases. Some difficulties also exist in treating brain metastases with drugs due to the difficulty of crossing the blood-brain barrier, which is the barrier between blood and brain tissue formed by brain capillaries and glial cell walls (8). However, drug therapy is important, especially in multiple brain metastases and progression after local treatment. Therefore, it is necessary to identify effective therapeutic drugs.

Researchers have conducted a series of clinical studies on the treatment of patients with HER2-positive breast cancer and brain metastases. For anti-HER2 treatment, drugs are mainly divided into large-molecule monoclonal antibodies, small-molecule tyrosine kinase inhibitor (TKI) drugs, and antibody-drug conjugate (ADC) drugs. The results of the tucatinib plus trastuzumab plus capecitabine regimen and the pyrotinib plus capecitabine regimen showed an improvement in overall response rate (ORR) in patients with HER2-positive breast cancer brain metastases (9, 10). The emergence of the ADC class combines large-molecule monoclonal antibodies with cytotoxic drugs to achieve cytotoxic drug targeting, thus precisely killing tumor cells (11–13). ADC drugs, such as trastuzumab deruxtecan and trastuzumab emtansine, significantly improved survival in patients with HER2-positive brain metastases (14). A phase II study of large-molecule monoclonal antibodies suggests that pertuzumab plus trastuzumab may have clinical value in some patients with HER2-positive brain metastases from breast cancer (15).

All of these drugs have shown high activity in treating patients with breast cancer brain metastases. However, it is unclear which treatment has the best effect. Therefore, we performed a meta-analysis of seven drugs currently used to treat HER2-positive breast cancer brain metastases to evaluate their effects and adverse effects.

## Methods

This systematic review was reported following the Preferred Reporting Items for Systematic Reviews and Meta-Analyses (PRISMA) guidelines (16).

### Search strategy and selection criteria

We conducted a systematic review and meta-analysis. We searched PubMed, Embase, and the Cochrane Library for randomized controlled trials (RCTs) and single-arm studies on the treatment of HER2-positive breast cancer brain metastases through May 20, 2022, and the last 5 years of the American Society of Clinical Oncology, European Society for Medical Oncology, and San Antonio Breast Cancer Symposium. We systematically searched the database using the following search terms: “HER2 positive breast cancer” or “HER2 positive breast carcinoma” or “HER2 positive breast neoplasms” and “recurrent or” or “advanced” or “metastatic” and “DS-8201” or “trastuzumab deruxtecan” or “T-Dxd” or “lapatinib” or “margetuximab” or “neratinib” “pertuzumab” or “pyrotinib” or “RC 48” or “disitamab vedotin” or “TDM-1” or “trastuzumab emtansine” or “trastuzumab” or “tucatinib.”

We used the following inclusion: criteria (1) inclusion in prospective phase II or III RCTs or single-arm studies; (2) inclusion of patients with HER2-positive breast cancer presenting with brain metastases, without differentiation between previous regimens and containing one or more of the 10 drug combinations described above; (3) hazard ratio (HR) values for included patients with ORR or progression-free survival (PFS) or OS and 95% confidence intervals (CIs). The exclusion criteria were as follows: (1) studies with survival data without brain metastases and (2) retrospective studies, meta-analyses, case reports, and reviews.

### Data extraction

Data on the characteristics of the studies were extracted by two independent investigators (XH and TW) and decided by discussion when disputes occurred. The main characteristics of the studies included in this study were the study name, study type, drug or protocol, follow-up time, and number of patients included. HRs and 95% CIs were extracted for ORR, PFS, and OS. All adverse events (AEs) and grade 3−5 AEs were extracted for anti-HER2 therapy. When multiple outcomes were reported, only the most recent ones were used.

### Statistical analysis

For the net meta-analysis of RCTs, we used the “Gemtc” package of the R version 4.1.2 software for network mapping and statistical analysis. The log and log standard errors of the HRs were calculated based on the HRs and 95% CIs. We used fixed-effects models to build the working models and analyze the data. To assess drug efficacy, we applied surface area under the cumulative ranking curve (SUCRA) values ranging from 0 to 1, with SUCRA values close to 1 indicating better drug efficacy.

Based on a single-group rate meta-analysis approach, we drew forest plots of ORR values for single-arm studies using Stata 14.0 to summarize and visualize the data. We also summarized the AEs of these drugs using a single-group rate meta-analysis approach. This was performed to provide a clearer picture of the data to the investigators.

The quality of the studies was assessed using the Cochrane collaboration tool. Consequently, each study was classified as high-risk, low-risk, or unclear. Data extraction and quality assessment were performed by two independent investigators, and disagreements were resolved by a third investigator upon notification (**Supplementary Table S1**).

## Results

The inclusion and exclusion search of the literature resulted in a total of 18,125 relevant studies, and after excluding 18,115 studies according to the inclusion and exclusion criteria, we finally included three RCTs and seven single-arm clinical studies (**Supplement Figure S1**) that involved a total of 731 patients with HER2-positive breast cancer brain metastases who were treated with trastuzumab deruxtecan, trastuzumab emtansine, lapatinib neratinib, pyrotinib, pertuzumab, and trastuzumab anti-HER2 agents. The physician’s choice group included chemotherapy (any single agent), hormonal therapy for hormone receptor-positive disease (single agent or dual therapy), or HER2-directed therapy (single agent, dual HER2-targeted therapy, or HER2-directed therapy combined with single-agent chemotherapy or single agent). The characteristics of the ten studies are summarized in **Table 1**.

**Table 1:** Type of trial and baseline characteristics.

Type	Study	Auther	Time	Phase	Treatment options	ORR (%)	Patients (*N*)	PFS (HR, 95% CI)
RCT	DESTINY-Breast03	G. Curigliano	2022	III	Trastuzumab deruxtecan	–	43	0.38 (0.23 -0.64)
					Trastuzumab emtansine	–	39	
	EMILIA	I. E. Krop	2015	III	Trastuzumab emtansine	–	45	1.00 (0.54 - 1.84)
					Lapatinib plus capecitabine	–	50	
	TH3RESA	Ian E Krop	2014	III	Trastuzumab emtansine	–	44	0·47 (0·24–0.89)
					Physician’s choice	–	28	
Single-arm study	DESTINY-Breast01	G. Jerusalem	2020	II	Trastuzumab deruxtecan	58.3	24	–
	TUXEDO-1	R. Bartsch	2021	II	Trastuzumab deruxtecan	73.3	15	–
	TBCRC 022	Rachel A	2019	II	Neratinib plus capecitabine	49	37	–
					Lapatinib plus capecitabine	33	12	
	PERMEATE	Min Yan	2022	II	Pyrotinib plus capecitabine ^a^	74.6	59	–
					Pyrotinib plus capecitabine ^b^	42.1	19	
	Lin.	Nancy U. Lin	2009	II	Lapatinib ^c^	6.4	94	–
					Lapatinib ^d^	6.3	143	
	LANDSCAPE	Thomas Bachelot	2013	II	Lapatinib plus capecitabine	57.1	42	–
	PATRICIA	Nancy U. Lin	2021	II	Pertuzumab plus trastuzumab	11	37	–

RCT: randomized controlled trial ORR: objective response rate PFS: progression free survival HR: hazard ratio Trastuzumab deruxtecan: DS-8201 Trastuzumab emtansine: T-DM1 N: number

a: Pyrotinib plus Capecitabine without previous radiotherapy. b: Pyrotinib plus Capecitabine progressive CNS disease after whole-brain radiotherapy. c: Eastern Cooperative Group performance status (PS) 0 to 1 and 1 or 2prior trastuzumab regimens. d: Eastern Cooperative Group PS 2 and/or >2 prior trastuzumab regimens.

### Network meta‐analysis of RCTs

**Figure 1:**
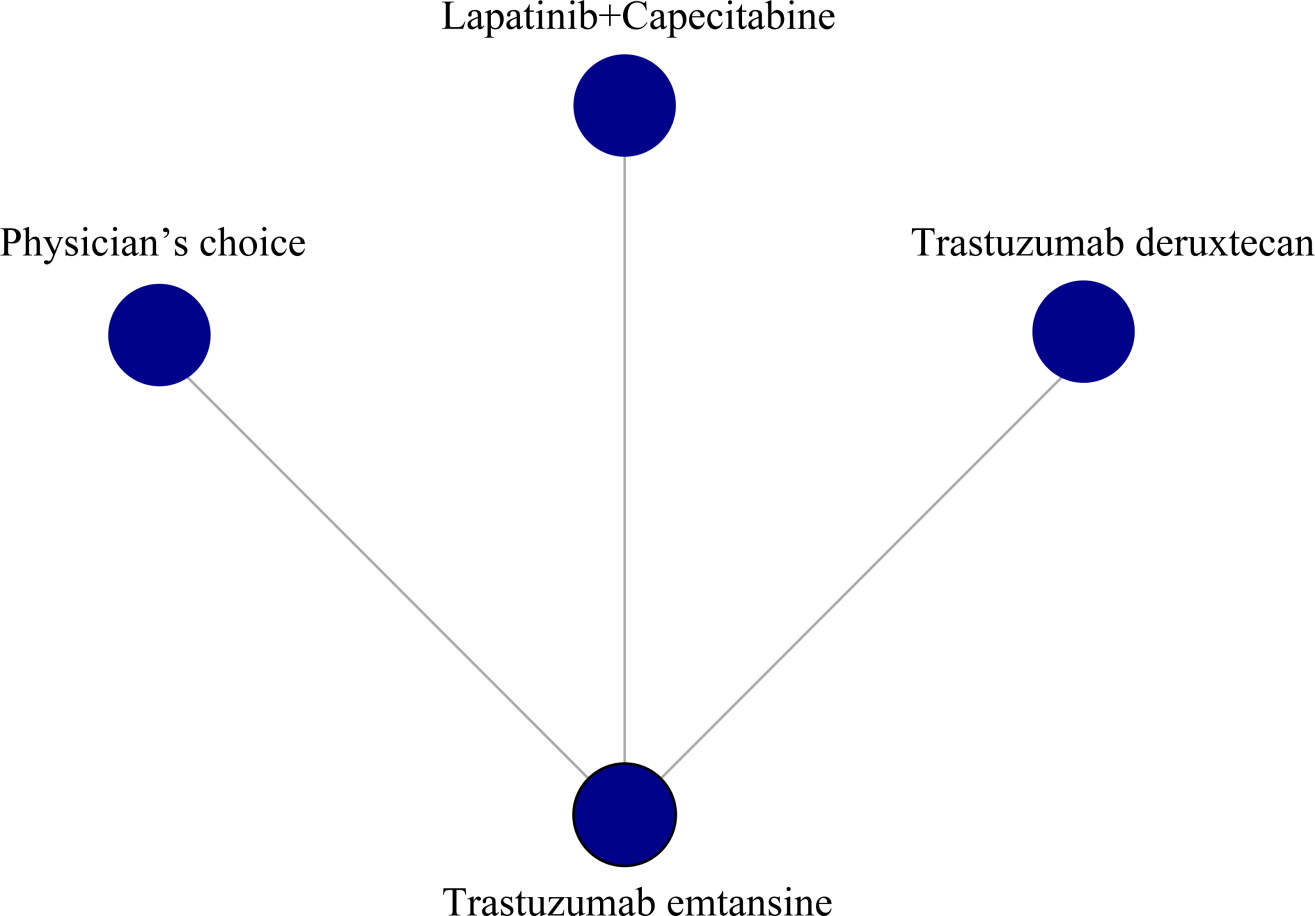
Network meta-analysis of progression-free survival.

We performed a network meta-analysis of the PFS of three RCTs, including a total of four treatment measures (trastuzumab deruxtecan, trastuzumab emtansine, lapatinib plus capecitabine, and physician’s choice group) for a total of 249 patients, with two monotherapy treatments and one combination treatment. Of all treatments compared, trastuzumab emtansine was the most commonly used drug (**Figure 1**).

**Figure 2:**
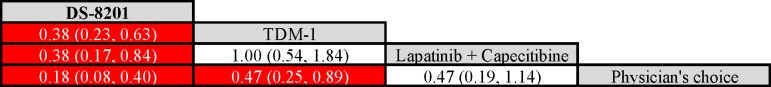
League table of network meta-analysis of the four anti-HER2 regimens. Red represents a statistically significant difference (*P* < 0.05). HER2, Human Epidermal Growth Factor 2. Trastuzumab deruxtecan, DS-8201; Trastuzumab emtansine, T-DM1.

**Figure 3:**
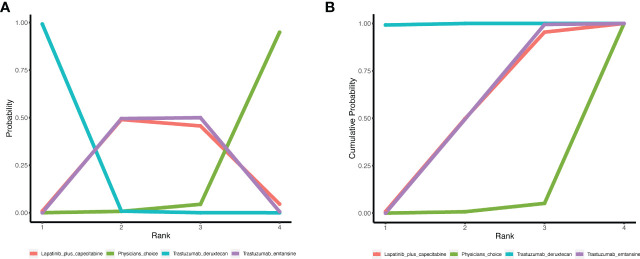
The ranking of PFS in anti-HER2 treatment regimens. **(A)** Individual ranking plots of PFS between different anti-HER2 treatment regimens. **(B)** The results of SUCRA for PFS between different anti-HER2 treatment regimens. PFS, progression-free survival; HER2, human epidermal growth factor 2; SUCRA, cumulative ranking curve.

The surface under SUCRA showed that trastuzumab deruxtecan had the highest efficiency (99.72%), followed by trastuzumab emtansine (49.66%) and lapatinib plus capecitabine (48.71%). The SUCRA for PFS according to the treatment regimen is shown in **Table 2**. In the league table, we found that trastuzumab deruxtecan was significantly superior to several other types of drugs or treatment strategies, while trastuzumab emtansine was second only to trastuzumab deruxtecan and superior to lapatinib plus capecitabine and the physician’s choice group (**Figure 2**). We observed the best effect of trastuzumab deruxtecan in individual ranking plots and the best effect in the cumulative ranking plots, with the largest area under the curve in the trastuzumab deruxtecan group. We also observed that the percentage of trastuzumab emtansine and lapatinib plus capecitabine is very close to each other (**Figure 3**).

**Table 2:** The cumulative ranking curve of each treatment option for progression-free survival.

Treatment	SUCRA(%)
Trastuzumab deruxtecan	99.72
Trastuzumab emtansine	49.66
Lapatinib + Capecitabine	48.71
Physician’s choice	1.90

SUCRA: umulative ranking curve. Trastuzumab deruxtecan: DS-8201.

Trastuzumab emtansine: T-DM1.

**Figure 4:**
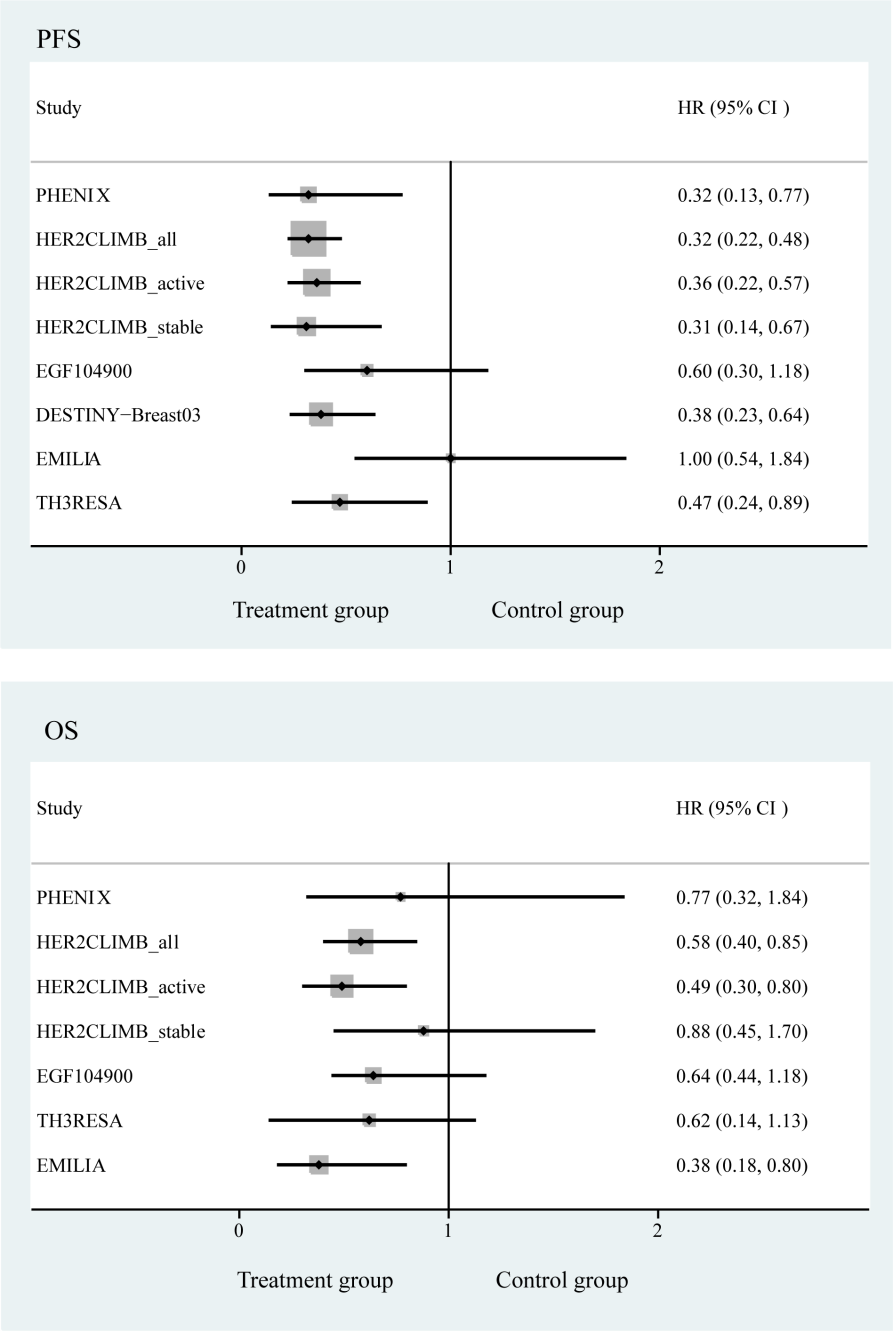
Forest plot of progression-free survival and overall survival in randomized controlled trials.

We pooled the survival of patients with brain metastases and observed that the regimens used in the DESTINY-Breast03, PHENIX, HER2CLIMB, and TH3RESA studies significantly improved PFS of the patients, with the most significant improvement in the DESTINY-Breast03 study (HR, 0.38; 95% CI, 0.23−0.64). We also examined the OS of patients with brain metastases and found that the regimens used in the HER2CLIMB_all and HER2CLIMB_active subgroups improved the OS of the patients, with a significant improvement in the HER2CLIMB_active subgroup (HR, 0.49; 95% CI, 0.30−0.80) (**Figure 4**).

### Single-arm studies

**Figure 5:**
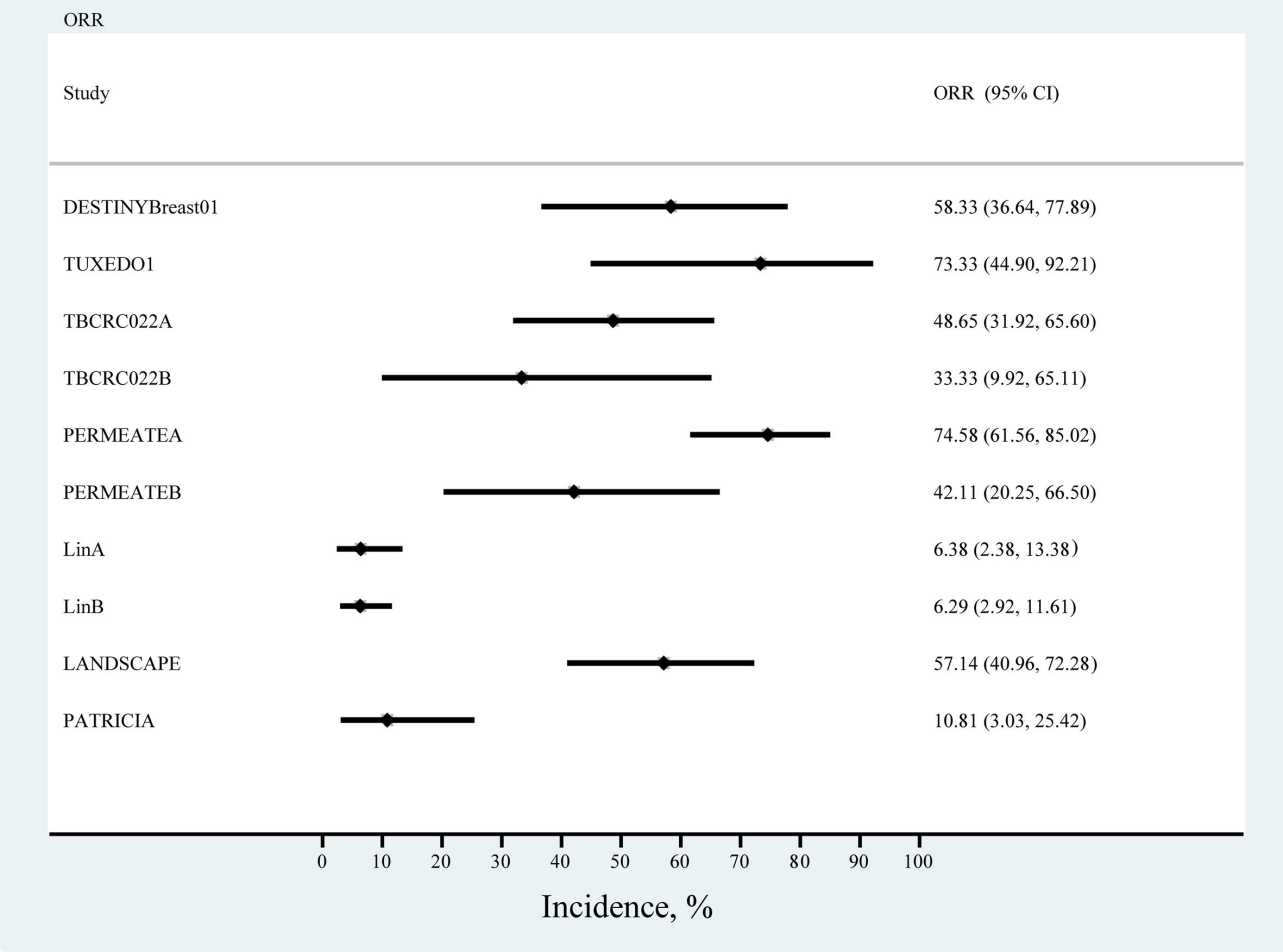
Forest plot of the overall response rate in single-arm studies.

We primarily analyzed ORR in seven single-arm studies, and the results of the meta-analysis showed the highest ORR rates for TUXEDO1 and PERMEATEA, with trastuzumab deruxtecan (ORR, 73.33%; 95% CI, 44.90%−92.21%) and pyrotinib plus capecitabine (ORR, 74.58%; 95% CI, 61.56%−85.02%). The lowest ORR was observed with lapatinib in the study by LinA. (ORR, 6.38%; 95% CI, 2.38%−13.38) (**Figure 5**).

### Drug-related adverse reactions

**Figure 6:**
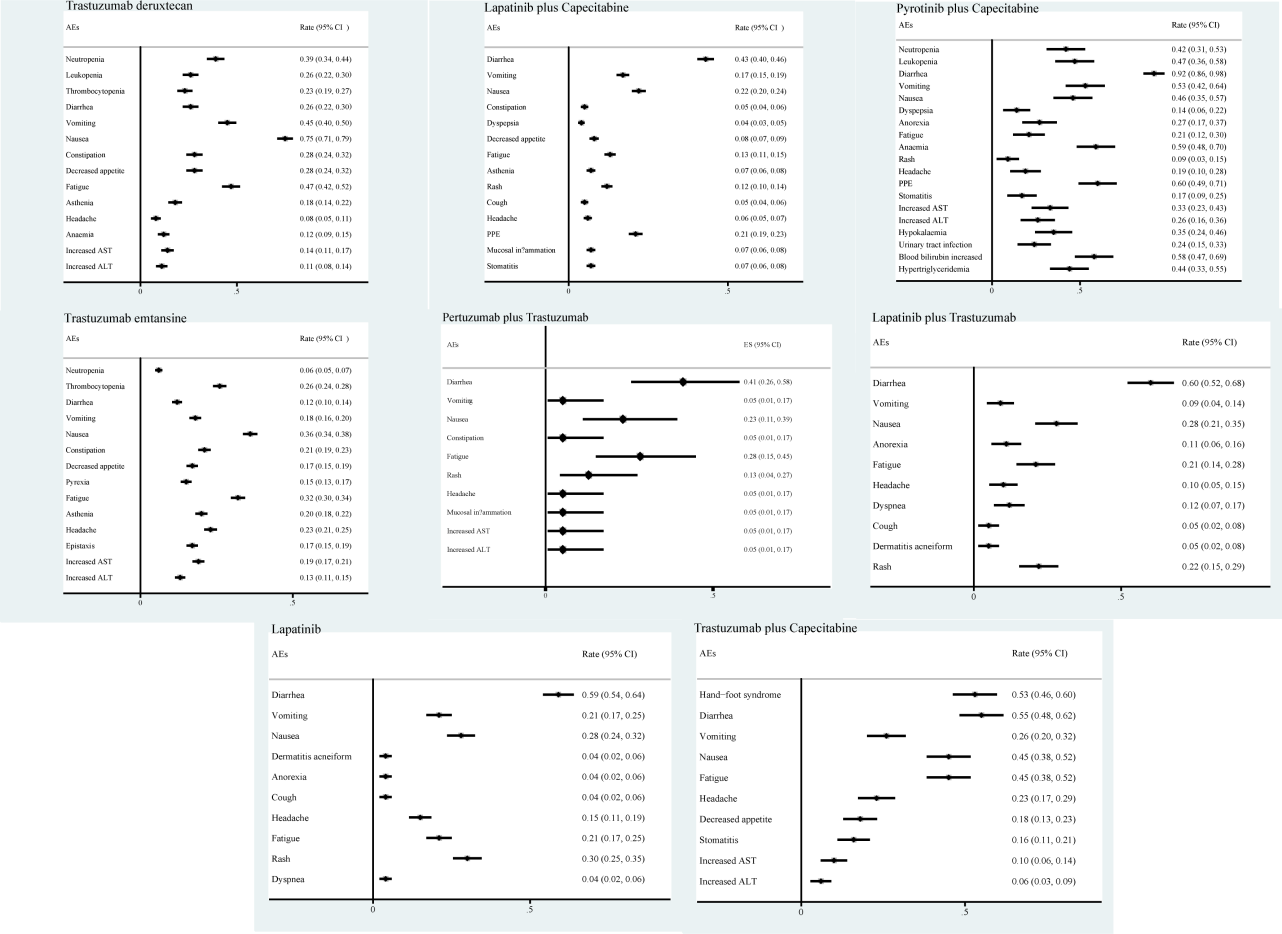
Adverse events associated with anti-Human Epidermal Growth Factor 2 therapeutic agents.

We also analyzed the incidence of AEs associated with eight drugs, monotherapy or combination regimens. The main AEs during treatment with trastuzumab deruxtecan drugs were nausea (75%), vomiting (45%), and fatigue (47%). With lapatinib plus capecitabine, pyrotinib plus capecitabine, pertuzumab plus trastuzumab, lapatinib plus trastuzumab, and lapatinib monotherapy regimens, the AEs that occurred were mainly diarrhea (43%, 92%, 41%, 60%, 59% respectively). The main AEs associated with trastuzumab emtansine treatment were nausea and fatigue. The main AEs of treatment with trastuzumab plus capecitabine were diarrhea (55%) and hand-foot syndrome (53%) (**Figure 6**).

## Discussion

To the best of our knowledge, this meta-analysis is the most comprehensive and the first to evaluate optimal treatment options for patients with HER2-positive breast cancer brain metastases. We analyzed seven anti-HER2 agents in patients with brain metastases from breast cancer and found that trastuzumab deruxtecan significantly improved PFS in patients in the RCT study, while trastuzumab deruxtecan significantly improved ORR in patients in the single-arm study. The overall AEs associated with the drugs were manageable.

The occurrence of brain metastases in patients with HER2-positive breast cancer is an important factor affecting patient survival, and considerable efficacy has been achieved with current drug treatments, including large-molecule monoclonal antibodies, ADCs, and small-molecule TKI drugs. Trastuzumab deruxtecan and pyrotinib are currently the most efficacious drugs in the latest clinical studies for the treatment of patients with brain metastases from breast cancer. Trastuzumab deruxtecan (DS-8201, T-Dxd) is an antibody-drug coupling drug consisting of an anti-HER2 antibody, a cleavable tetrapeptide linker, and a cytotoxic topoisomerase I inhibitor to achieve precise tumor cell killing (17). Pyrotinib inhibits tumor cell growth by blocking the formation of homodimers and heterodimers of EGFR and HER2 in tumor cells, inhibiting their phosphorylation, and blocking the activation of downstream signaling pathways (9).

Our network meta-analysis based on RCTs showed that trastuzumab emtansine was the drug compared most frequently, and among several anti-HER2 regimens for the treatment of patients with brain metastases from breast cancer, trastuzumab deruxtecan was the most effective. In the DESTINY-Breast03 study (14), the use of trastuzumab deruxtecan significantly improved PFS and OS in patients compared to trastuzumab emtansine in a subgroup analysis of stable brain metastases; trastuzumab deruxtecan was significantly better than the control group in both subgroup analyses of stable brain metastases. The results of the current DESTINY-Breast01 and DESTINY-Breast03 studies show that trastuzumab deruxtecan significantly prolongs survival in patients with brain metastases from breast cancer for multiple lines of therapy beyond the second line in advanced HER2-positive breast cancer, especially in patients with advanced breast cancer who have failed multiple lines of therapy. Therefore, trastuzumab deruxtecan can be placed in second-line treatment if there are other treatment options that can still achieve good results in terms of cost effectiveness and actual benefit of treatment (18, 19). ADC drugs improve survival not only in HER2-positive breast cancer patients, but also in patients with low expression of HER2. HER2-low-expressing breast cancer is a heterogeneous group of HER2-low-expressing population (20, 21). The results of the DESTINY-Breast04 study showed that compared to the physician-selected treatment group, patients on trastuzumab deruxtecan with low expression of HER2 showed unprecedented statistically significant and clinically meaningful improvements in PFS and OS. Therefore, the DESTINY-Breast04 study identified MBC with low expression of HER2 (immunohistochemistry (IHC) 1+, IHC 2+/fluorescence *in situ* hybridization (FISH)-) as a new target group of patients and trastuzumab deruxtecan as a new standard of care; similarly, the survival outcomes of patients with brain metastases in this study were more promising (22). The ongoing DESTINY-Breast09 studies, which include a HER2-low expression population and more Chinese first-line patients, are promising.

Some randomized controlled studies were not included in the network meta-analysis due to their lack of comparison. We initially searched for RCTs including the drugs “margatuximab” or “tucatinib”, but we found that (e.g., the HER2CLIMB study) these studies could not be included in the network, so we excluded studies with these drugs. However, in order to summarize the anti-her2 treatment in breast cancer patients with brain metastases. Therefore, we summarized the PFS and OS of these randomized controlled studies using a forest plot. In the HER2CLIMB study, we found that the combination regimen with the addition of tucatinib significantly improved PFS in each subgroup compared to trastuzumab plus capecitabine (10, 23). However, these studies did not report an improvement in OS in patients with stable brain metastases. Therefore, this is an important conclusion. Tucatinib plus trastuzumab plus capecitabine is recommended for patients with active brain metastases. However, there are no randomized controlled studies on ADC drugs, and their comparative efficacy is unclear. In addition, there are fewer RCTs of anti-HER2 therapy for the HER2-positive breast cancer brain metastasis population and the experimental and control groups are different, and we used a network approach that still cannot fully accommodate these shortcomings. However, this study clarifies the current preferred drug recommendations for the HER2-positive breast cancer brain metastasis population by summarizing and comparing the available studies. Moreover, we found that the therapeutic effects of trastuzumab emtansine and lapatinib plus capecitabine were very similar. Based on this result, lapatinib plus capecitabine was more friendly to patients with brain metastases compared to trastuzumab emtansine in terms of affordability and drug accessibility. The addition of more RCTs by future scientific groups may influence these data and percentages.

Several single-arm studies are available to treat breast cancer with brain metastases. Therefore, we also summarized the ORR rates of current single-arm studies. Overall, the TUXEDO1 and PERMEATE studies reported the highest ORR rates in cohort A (9, 24). Treatment regimens were trastuzumab deruxtecan and pyrotinib plus capecitabine, thus concluding that both are very good systemic treatments for HER2-positive breast cancer brain metastases. One hypothesis suggests that the blood-brain barrier breaks after radiotherapy for intracranial lesions, making it easier for drugs to penetrate. Another hypothesis suggests that radiotherapy destroys the surrounding blood vessels, which results in a relative decrease in drug permeability and a decrease in the efficiency of systemic therapy. Based on the two opposing hypotheses, patients with active brain metastases from HER2-positive breast cancer in the PERMEATE study cohort A were patients without radiotherapy and cohort B were patients who progressed after radiotherapy. The results of the study clearly showed that the ORR was much lower in patients who progressed after radiotherapy than in those who did not. Therefore, the latter hypothesis may be more credible.

We also summarized the AEs of different regimens. Interestingly, we found that the highest incidence of AE for most small-molecule TKI drugs was diarrhea, while for ADC drugs represented by trastuzumab deruxtecan and trastuzumab emtansine, the main symptoms were nausea, vomiting, and fatigue. Therefore, we considered that the AEs of small-molecule TKI drugs are primarily gastrointestinal reactions, such as diarrhea, while the AEs of ADC drugs are mainly focused on the AEs of chemotherapeutic drugs. However, some side effects not included in our study also deserve attention, such as interstitial pneumonia caused by trastuzumab deruxtecan drugs and platelet decrease caused by trastuzumab emtansine drugs. Therefore, knowing AEs is essential for the application of drugs to treat patients with HER2-positive breast cancer brain metastases and must be intervened or prevented in advance.

This meta-analysis has some limitations. First, it was a meta-analysis based on literature rather than individual patient data, and there were differences in protocols and evaluation criteria between studies. Second, only three randomized controlled studies were included in this study, which is a small number. Third, due to differences in follow-up time, in the single-arm study, only the ORR of the patients was analyzed.

## Conclusion

In an RCT study, treatment with trastuzumab deruxtecan significantly improved patient survival in HER2-positive patients with brain metastases from breast cancer. Small-molecule TKI drugs, represented by the pyrotinib plus capecitabine regimen, also achieved excellent ORR rates in single-arm studies. We found that the main side effects of ADC drugs were nausea and fatigue, while the main side effects of TKI drugs and large monoclonal antibodies were diarrhea.

## Data availability statement

The original contributions presented in the study are included in the article/**Supplementary Material**. Further inquiries can be directed to the corresponding author.

## Author contributions

XH, JZ contributed to the conception and the drafting of manuscripts. FZ, DR are responsible for coordinating and participating in the article revision. All authors read and approved the final manuscript. XH, GS, and TW contributed equally to this work and are co–first authors. Concept and design: XH, MW, JZ. Acquisition, analysis, or interpretation of data: XH, JL. Drafting of the manuscript: XH, JZ. Critical revision of the manuscript for important intellectual content: All authors. Statistical analysis: XH, FZ. Obtained funding: All authors. Administrative, technical, or material support: All authors. Supervision: All authors. All authors contributed to the article and approved the submitted version.
