# Supplementary material for: Treatment options for patients with human epidermal growth factor 2-positive breast cancer brain metastases: A systematic review and meta-analysis
Source: Front Oncol. 2023 Feb 20;13:1003565. doi: 10.3389/fonc.2023.1003565 (PMC9986525; doi:10.3389/fonc.2023.1003565)
Supplement: Supplementary file 1 [file DataSheet_1.pdf]

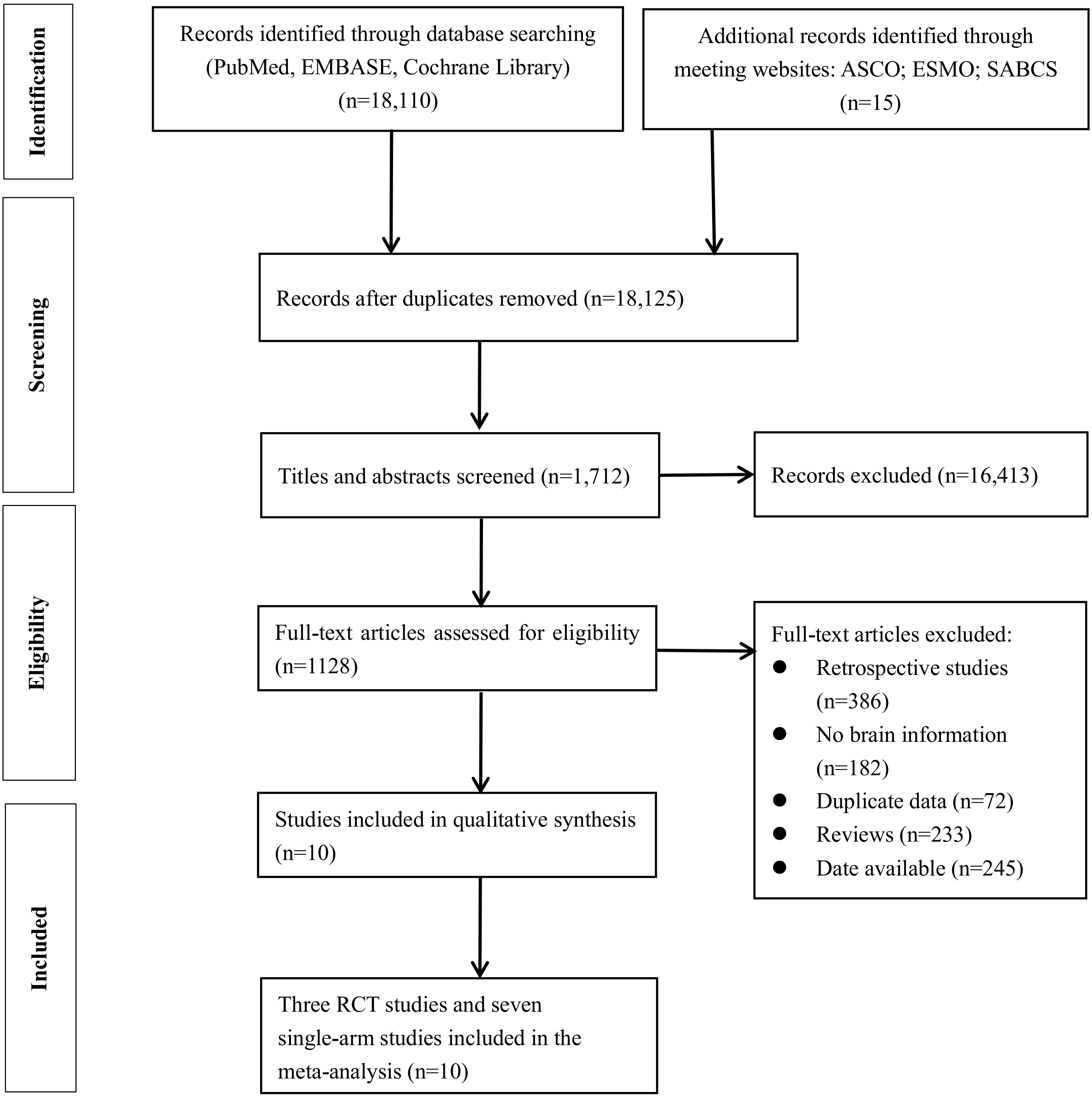

ASCO, American Society of Clinical Oncology; ESMO, European Society for Medical Oncology, SABCS, San Antonio Breast Cancer Symposium; RCT, Randomized controlled trials.

Figure S1. Search string and flow charts for filtering and research selection.

Study	Year	Randomization	Allocation concealment	Blinding	Incomplete outcome data	Selective reporting	Other sources of bias
DESTINY-Breast03	2022	Low	High	Low	Low	Low	Low
EMILIA	2015	Low	High	Low	Low	Low	Low
TH3RESA	2014	Low	High	Low	Low	Low	Low

Table S1. Assessment of bias of randomized controlled trials.
